# Screening of visuospatial abilities in amyotrophic lateral sclerosis (ALS): a pilot study using the battery for visuospatial abilities (BVA)

**DOI:** 10.1186/s13023-025-03645-z

**Published:** 2025-03-08

**Authors:** Minoo Sharbafshaaer, Mattia Siciliano, Carla Passaniti, Valeria Sant’Elia, Marcello Silvestro, Antonio Russo, Sabrina Esposito, Gioacchino Tedeschi, Luigi Trojano, Francesca Trojsi

**Affiliations:** 1 Department of Advanced Medical and Surgical Sciences, MRI Research Center, Università degli Studi della Campania “Luigi Vanvitelli”, Naples, Italy; 2 First Division of Neurology, AOU Università degli Studi della Campania “Luigi Vanvitelli”, Naples, 80138 Italy; 3 Department of Psychology, Università degli Studi della Campania “Luigi Vanvitelli”, Caserta, Italy; 4 Neurosciences Research Centre, Molecular and Clinical Sciences Research Institute, St George’s University of London, London, UK

**Keywords:** Amyotrophic lateral sclerosis, Neuropsychology, Cognitive assessment, Spatial cognition, Battery for visuospatial abilities (BVA), ECAS, Pilot study, Neurodegenerative diseases

## Abstract

**Background:**

Cognitive deficits related to frontotemporal dysfunction are common in Amyotrophic Lateral Sclerosis (ALS). Visuospatial deficits, related to posterior cerebral regions, are often underestimated in ALS, though they play a crucial role in attending daily living activities. Our pilot study aims at assessing visuospatial abilities using a domain-specific tool in ALS patients compared to healthy controls (HC).

**Methods:**

Twenty-three patients with early ALS and 23 age- and education-matched HC underwent the Battery for Visuospatial Abilities (BVA), including 4 visuo-perceptual and 4 visuo-representational subtests.

**Results:**

When compared to HC, ALS scored worse in 2 visuo-perceptual subtests (i.e., Line Length Judgment and Line Orientation Judgment) and 1 visuo-representational tasks (i.e., Hidden Figure Identification, HFI) (*p* < 0.01). No correlations arose between ALS clinical features and BVA performance. More than 80% of the ALS cohort obtained abnormal scores in the HFI subtest.

**Conclusions:**

Our findings revealed that patients with ALS scored worse (compared to HC) on selective tests tapping “perceptual” and “representational” visuospatial abilities, since the early stages of disease. In clinical practice, our findings highlight the need for multi-domain neuropsychological assessment, for monitoring disease courses and properly organizing care management of patients with ALS.

**Supplementary Information:**

The online version contains supplementary material available at 10.1186/s13023-025-03645-z.

## Introduction

Amyotrophic lateral sclerosis (ALS) is an adult-onset motor neuron and multisystem disease [1] that is mainly characterized by progressive motor symptoms, such as muscle weakness, muscle atrophy, and spasticity. Over the past twenty years, several clinical studies have highlighted that clinical presentation in ALS can be quite heterogeneous [2, 3]. Up to 50% of ALS cases are first identified with cognitive dysfunctions [4], which may worsen and show different profiles across stages of the disease [5–9]. Executive and behavioral dysfunctions may have prognostic implications [10, 11]. Moreover, in ALS, motor and cognitive components appear to worsen in parallel, especially when the bulbar function is involved [8, 12]. Notably, advanced neuroimaging studies revealed widespread damage to extra-motor networks underlying cognitive and behavioral functions during disease progression [13–15]. Consequently, ALS-specific cognitive and behavioral impairments are more frequent in more advanced disease stages [7, 9]. However, cognitive and motor involvement may present distinct trajectories across the disease course, suggesting a differential vulnerability of motor and non-motor cortical networks in different disease phenotypes [5, 13]. Increasing evidence suggests that cognitive and behavioral impairment in ALS over-laps with pathological and genetic features, as TDP-43 pathologic burden has been associated with cognitive impairment [16, 17] and C9orf72 repeat expansion has been revealed in ALS patients with rapid cognitive decline and poor survival [18, 19].

The cognitive profile typically associated with amyotrophic lateral sclerosis (ALS) is characterized by deficits in verbal fluency, language, social cognition, and executive functions [4]. However, visuospatial abilities, encompassing both visuo-perceptual and representational components, are not systematically assessed in this population. For instance, the Edinburgh Cognitive and Behavioural Amyotrophic Lateral Sclerosis Screen (ECAS) [20], a widely used assessment tool, does not comprehensively evaluate these cognitive domains. In contrast, the Battery for Visuospatial Abilities (BVA) offers a more precise and targeted approach to diagnosing and monitoring visuospatial deficits. The BVA’s specialized focus on visuospatial skills, combined with its detailed normative data, sensitivity to subtle deficits, and applicability to rehabilitation, positions it as a more effective tool for assessing and managing visuospatial impairments in clinical and research settings.

Evidence in ALS suggested that the visuo-representational and the visuo-perceptual abilities play a crucial role in managing activities of daily living and in preserving patients’ well-being [21], such as in spatial orientation mediated by environmental cues [22]. Moreover, visuo-representational, and visuo-perceptual abilities participate in generating, retaining, and transforming visual images [23], processing the overall configuration of perceptual stimuli, appreciating their position, and performing mental operations on their spatial representation [24]. In terms of neural correlates, visuo-representational and visuo-perceptual functions are mediated by a wide, distributed neural network including the parietal lobes, the lateral prefrontal cortex, the medial temporal lobes, the inferior temporal cortex, the occipital cortex, and the basal ganglia, particularly in the right hemisphere [24].

A useful battery to explore both the perceptual component and the representational component of visuospatial abilities, independent from motor impairment, is the BVA, known in Italy as TeRaDiC; [25–27]), available in English and Italian. Yet, to date, no study applied the BVA in the ALS.

The present pilot study aims to fill the literature gap on the impairment of visuospatial abilities in ALS by means of the 8 subtests included in the BVA. We hypothesize that patients with ALS exhibit marked deficits in both visuo-perceptual and visuo-representational tasks, as evaluated by the BVA, compared to healthy controls. Early detection of these impairments may contribute to more effective clinical management by enabling tailored interventions and comprehensive monitoring of disease progression.

## Materials and methods

### Participants

Twenty-three right-handed patients with definite or clinical/laboratory-supported probable ALS, according to the El-Escorial revised criteria [28], showing classic (*n* = 7), bulbar (*n* = 2), flail limbs (*n* = 11) and 3 pyramidal phenotypes [2], were included. These patients were prospectively recruited across the First Division of Neurology of the University of Campania “Luigi Vanvitelli” (Naples, Italy) between December 2021 and April 2022. Exclusion criteria were a history of other neurologic or psychological conditions, and alcohol or drug addiction. Genetic analysis was performed in all patients, exploring *C9orf72* repeat expansion and mutations of *SOD1*, *TARDBP*, and *FUS/TLS*. No mutations of these genes were reported.

Twenty-three age- and education-matched healthy adults were additionally recruited as healthy controls (HC) group through research volunteer panels held by the First Division of Neurology of “Luigi Vanvitelli” University (Naples, Italy), non–blood caregivers of patients with ALS, and local community noticeboards.

For all subjects, exclusion criteria were medical illnesses or substance abuse that could interfere with cognitive functioning; any other major systemic, psychiatric, or neurological diseases, including dementia; other causes of brain damage, including lacunae and extensive cerebrovascular disorders; and a vital capacity lower than 70% of the predicted value.

Procedural consistency was ensured by following a structured order of subtests for all participants, as described in Trojano et al. [27]. This non-randomized approach ensured consistency and uniformity in task administration. The assessments were conducted by a trained examiner who followed standardized protocols to minimize possible sources of variability. Furthermore, the examiner was blinded to the study hypotheses and was trained to strictly adhere to the predefined evaluation protocols and guidelines outlined in Trojano et al. [27]. These measures ensured objectivity and consistency in the assessment process across all participants.

Ethics approval was obtained from the Ethics Committee of the University of Campania “Luigi Vanvitelli” in Naples, Italy (Protocol nr. 591/2018). According to the Declaration of Helsinki, all participants provided informed consent to participate in the study.

### Materials

ALSFRS-R: the ALSFRS-R is a disease-specific 12-item tool assessing patients’ functional abilities to perform independent tasks. The questionnaire is structured on a 5-point scale ranging from 4 to 0, where 4 indicates no loss of function and 0 indicates total loss of function. The ALSFRS-R includes four scales, each measuring one domain affected by the disease [29].

ECAS: The ECAS is a short screening test (15–20 min) assessing cognitive impairment in ALS [20], providing sub-scores for language, fluency, executive, memory, and visuospatial abilities. Language is evaluated by naming, comprehension, and spelling. Fluency is measured by the free production of words beginning with the letter “s” and a restrained production of words beginning with the letter “t” but with only four letters. Executive functions are measured by a reverse digit span, alternation of letters and numbers, inhibitory sentence completion, and social cognition. The memory subscale includes measurements of immediate recall, delayed percentage retention, and delayed recognition. Finally, visuospatial abilities are measured with dot and cube counting, and number location.

BVA - perceptual subtests: this battery consisted of four tasks: each item is composed of a stimulus presented on the left and the four-choice display presented on the right [27]. Items are presented one at a time, and participants are required to point to the only item identical to the stimulus among the distracters without time constraints. Scoring procedures assign one point for each correct response; no penalty is computed for wrong responses. The first subtest is line length judgment (LLJ), in which participants are required to identify the line with the same length as the stimulus in the four-choice display. Item complexity increases during the task as linear differences between stimuli and distracters gradually decrease (score range: 0–20). The second subtest is line orientation judgment (LOJ), in which participants must identify, in the four-choice display, the line with the same orientation as the stimulus presented on the left side. The difference in orientation between stimulus and distracters is 30° in half of the items and 15° in the remaining trials. In the first seven items, distracters (the same length as the stimulus) are arranged as a sunburst, while in the last three items, distracters are randomly spread on the four-choice display (score range: 0–10). The third subtest is angle width judgment (AWJ), in which participants should identify the angle with the same width as the stimulus in the four-choice display. The distracters differ from 15° to 90° from the stimulus (score range: 0–10). The fourth subtest is pointing position identification (PPI): participants are required to identify the square with the same configuration of 1–3 embedded points as the stimulus. Distracters in the four-choice display have the same number of points as the stimulus but in different spatial arrangements (score range: 0–12).

BVA—representational subtests: The four tasks included in this section assess participants’ ability to mentally represent spatial relationships; three of them include a four-choice display, as above, and the last task has a different arrangement [27]. Each correct response is assigned one point. The first subtest is mental rotation: participants are required to mentally rotate bidimensional stimuli (the italic capital letter L or S, with small white or black circles at the extremities) on the horizontal plane and to identify the only item in the display matching it. The four-choice displays enclose the stimulus item, rotated by 45°, 90°, 135°, or 180°, together with three mirror forms of the stimulus (distracters), printed at different degrees of rotation. Prior to the task, participants receive two practice trials that can be solved with the aid of solid items (score range: 0–10). The second subtest is complex figure identification (CFI, shape recognition): participants have to identify the only figure matching the nonsense geometrical shape (not easily described verbally and of increasing complexity) presented on the left side in the four-choice display. Two practice trials are given before the task (score range: 0–10). The third subtest is hidden figure identification (HFI): stimuli consist of nonsense, complex geometrical patterns. Participants must disassemble each stimulus in their minds to identify, among the four complex geometrical patterns shown in the four-choice display, the only shape exactly embedded in the stimulus. Two practice trials are given (score range: 0–10). The fourth subtest is mental construction: in this task, participants must mentally assemble bidimensional stimuli. Stimuli consist of squares randomly subdivided into four parts, that are randomly shown on the right side of the display. In every trial, the examiner names two of these components, and participants must identify with which side they are contiguous in the stimulus (printed on the left side). Solid stimuli are used to explain the task in two practice trials. Two questions are foreseen for each trial; each correct response is scored 1 point (score range: 0–20).

### Statistical analyses

An a priori power analysis was performed using G*Power 3.1 with the following parameters: probability level (α): 0.05, statistical power (1 - β): 0.80, large effect size (Cohen’s d of 0.8 for Mann-Whitney test and rs of 0.5 for Spearman correlation analysis). According to Pitman, the required sample size for Spearman’s correlation analyses was determined by multiplying the sample size calculated for the equivalent parametric test (Pearson’s correlation test) by a correction factor. The results of the a priori power analyses indicated that a minimum of 42 individuals (i.e., 21 for each study group) were required for the Mann-Whitney test and 26 individuals for the Spearman’s correlation analysis to obtain a large effect size with a statistical power of 0.80 and an α level of 0.05. We used the Mann–Whitney test (U-test) or Pearson’s chi-squared test (χ2) to compare the patients with ALS and HC on demographics (i.e., age, education, and sex), BVA-perceptual subtests, and BVA-representational subtests. We employed Spearman’s correlation analyses to explore the associations between the clinical features (i.e., disease duration, ALSFRS-R, and UMN) and the accuracy in BVA subtests. Finally, we reported the percentage of ALS and HC with age- and education-adjusted scores in BVA subtests below normative data [27]. All multiple comparisons were corrected by the Bonferroni procedure, where the corrected p-value lower than 0.05 was considered statistically significant. All analyses were performed using the IBM Statistical Package for Social Science (SPSS) version 25 (Chicago, IL, USA).

## Results

Patients with ALS and HC did not differ in demographics (Table 1). Spearman’s correlation analyses did not show significant associations between ALS clinical features and the accuracy in visuo-perceptual and visuo-representational BVA subtests (Table 2). No data were missing or excluded.

**Table 1:** Descriptive statistics

Variables	ALS (*n* = 23)	HC (*n* = 23)	Mann-Whitney/χ^2^	*p*-value	Adj-*p*
*Demographics*:					
Age	64.00 [53.00, 69.00]	61.00 [53.00, 65.00]	231.50	0.468	1.000
Education, years	8.00 [5.00, 13.00]	8.00 [8.00, 13.00]	171.50	0.036	0.108
Sex (male)	17 (73.9%)	9 (39.1%)	5.66	0.017	0.051
*Clinical features*:					
Disease duration, months	35 [27.00, 46.00]	-	-	-	-
ALSFRS-R (total score)	29.00 [21.00, 37.00]	-	-	-	-
UMN score	7.00 [3.00, 10.00]	-	-	-	-
*Cognitive assessment*:					
MoCA	-	24.65 [23.65, 26.98]; 0.0%^a^	-	-	-
ECAS total score	96.48 [81.39, 103.01]; 4.3%^a^	-	-	-	-
ECAS sub-scores:					
Language	22.27 [17.94, 24.73]; 13.0%^a^	-	-	-	-
Verbal Fluency	17.88 [13.77, 22.20]; 8.7%^a^	-	-	-	-
Executive functions	29.15 [24.85, 32.35]; 13.0%^a^	-	-	-	-
Memory	15.84 [12.89, 18.07]; 8.7%^a^	-	-	-	-
Visuospatial abilities	11.54 [11.10, 12.16]; 4.3%^a^	-	-	-	-

Note. Data are reported as median [25th percentile, 75th percentile] or count (percentage); a percentage of patients or healthy controls with age- and education-adjusted score below normal population; Adj-p represents p-value corrected for multiple comparisons using the bonferroni procedure, and statistically significant differences are shown in **bold** while non-significant adjusted results are marked with *

**Abbreviations**: ALSFRS-R, amyotrophic lateral sclerosis functional rating Scale-Revised; UMN, upper motor neuron; ECAS, Edinburgh cognitive, and behavioural ALS screen

**Table 2:** Spearman’s correlations (*r*_s_) between clinical features and the accuracy in visuo-perceptual and visuo-representational tasks

	Disease duration	ALSFRS-*R*	UMN score
*BVA-perceptual tasks*:			
Line length judgment (LLJ)			
*r*_s_	-0.04	-0.04	0.20
*p*-value	0.872	0.847	0.368
Adj-*p*	1.000	1.000	1.000
Line orientation judgment (LOJ)			
*r*_s_	-0.16	0.02	0.25
*p*-value	0.469	0.921	0.256
Adj-*p*	1.000	1.000	1.000
Angle width judgment (AWJ)			
*r*_s_	-0.03	-0.05	0.01
*p*-value	0.885	0.831	0.968
Adj-*p*	1.000	1.000	1.000
Point position identification (PPI)			
*r*_s_	-0.20	-0.04	0.28
*p*-value	0.351	0.865	0.196
Adj-*p*	1.000	1.000	1.000
*BVA-representational tasks*:			
Mental rotation (MR)			
*r*_s_	0.13	-0.17	0.16
*p*-value	0.559	0.426	0.463
Adj-*p*	1.000	1.000	1.000
Complex figure identification (CFI)			
*r*_s_	-0.06	0.01	0.01
*p*-value	0.771	0.968	0.968
Adj-*p*	1.000	1.000	1.000
Hidden figure identification (HFI)			
*r*_s_	-0.07	0.08	0.33
*p*-value	0.752	0.726	0.128
Adj-*p*	1.000	1.000	1.000
Mental construction (MC)			
*r*_s_	-0.17	0.32	0.33
*p*-value	0.443	0.137	0.129
Adj-*p*	1.000	1.000	1.000

Note: Adj p-value represents p-value corrected for multiple comparisons using the Bonferroni procedure and statistically significant correlations are shown in **bold**, while non-significant adjusted results are marked with *. Spearman’s correlation coefficients (r_s_) interpretation: r_s_ ≈|0.10| small effect; r_s_ ≈|0.30| moderate effect; r_s_ ≈|0.50| large effect; Spearman’s correlation coefficients (r_s_) at least higher than|0.30| were reported in *italics*

Abbreviations: ALSFRS-R, Amyotrophic Lateral Sclerosis Functional Rating Scale-Revised; UMN, Upper Motor Neuron.

**Fig. 1:**
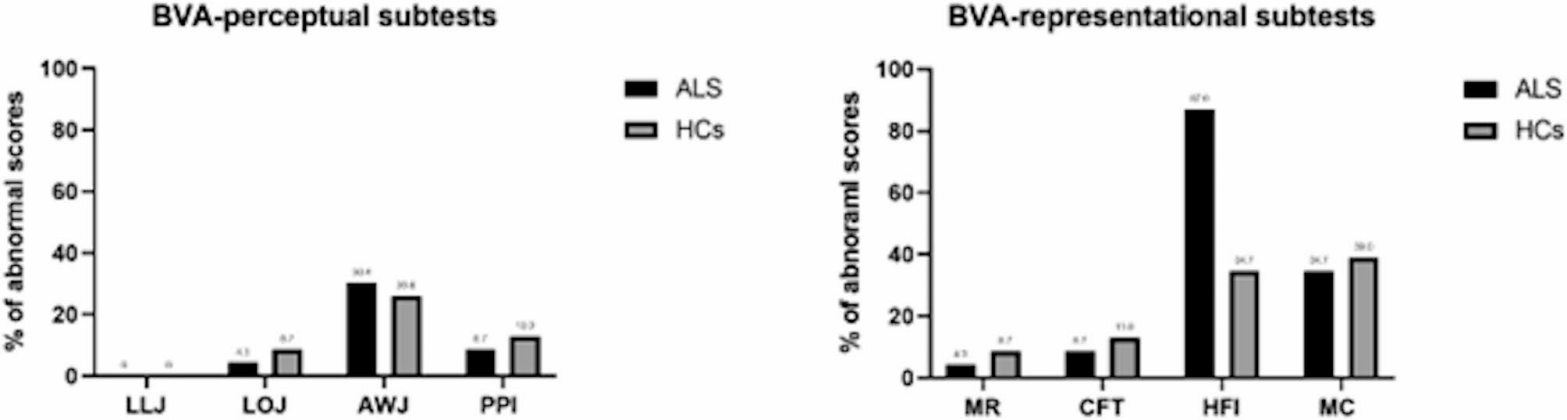
Percentage of ALS and healthy controls with age- and education-adjusted scores in BVA subtests below normative data

Compared with HC, the ALS group performed worse on the BVA perceptual LLJ and LOJ subtests and the BVA-representational HFI subtests (Table 3; the analysis considered the age- and education-adjusted BVA scores). Since deficits in language or executive functions could impact these results, we also ran Mann–Whitney analyses considering only the subgroup of ALS free from impairments in executive functions and/or language disturbances (as assessed on ECAS, *n* = 19), which basically confirmed the pattern above (see Supplementary Material 1). In particular, the percentage of patients with pathological scores on cognitive measures were: 4.3% for ECAS total score, 13% for ECAS language score, 8.7% for ECAS verbal fluency, 13% for ECAS executive functions, 8.7% for ECAS memory and 4.3% for ECAS visuospatial ability. Figure 1 reported the percentage of abnormal scores in BVA tasks for ALS and HC.

**Table 3:** Comparison between amyotrophic lateral sclerosis (ALS) and healthy controls (HC) in age- and education-adjusted perceptive and representational tasks of BVA

	ALS (*n* = 23)	HC (*n* = 23)	Mann-Whitney	*p*-value	Adj *p*
*BVA-perceptual tasks*:					
Line length judgment (LLJ)	15.82 [14.80, 17.03]	18.66 [16.77, 19.06]	84.50	< 0.001	**< 0.001**
Line orientation judgment (LOJ)	6.22 [4.77, 7.48]	8.55 [6.96, 9.27]	137.00	0.005	**0.020**
Angle width judgment (AWJ)	2.07 [0.81, 4.33]	4.90 [1.06, 5.46]	155.00	0.016	0.128
Point position identification (PPI)	8.22 [7.69, 8.93]	8.68 [7.02, 9.70]	263.00	0.974	1.000
*BVA-representational tasks*:					
Mental rotation (MR)	8.39 [5.34, 8.78]	6.78 [3.30, 9.28]	215.00	0.277	1.000
Complex figure identification (CFI)	8.09 [7.61, 8.43]	7.76 [6.85, 8.22]	208.50	0.219	1.000
Hidden figure identification (HFI)	0.00 [0.00, 1.61]	3.52 [0.30, 5.22]	108.00	< 0.001	**< 0.001**
Mental construction (MC)	9.52 [4.24, 11.24]	9.11 [5.18, 9.96]	235.00	0.517	1.000

Note: BVA scores refer to demographically adjusted scores according to Trojano et al.’s normative data. Pearson’s *r* interpretation: *r* ≈|0.10| small effect; *r* ≈|0.30| moderate effect; *r* ≈|0.50| large effect; 95%CI, 95% confidence interval of p-value based on Monte Carlo simulations with 10.000 repetitions; U-test, Mann-Whitney test; data are reported as median [25th percentile, 75th percentile] or count (percentage); adj p-value represents p-value corrected for multiple comparisons using the Bonferroni procedure and statistically significant differences using this procedure are shown in **bold**, while non-significant adjusted results are marked with *

## Discussion

In the present study, we systematically assessed the visuospatial abilities of ALS patients using the BVA, a battery designed to mitigate the influence of motor impairments by incorporating simple pointing or verbal responses, as preferred by the patients. Our results indicate that patients with ALS performed significantly worse than healthy controls (HCs) on two visuo-perceptual tasks - LLJ and LOJ - as well as on one visual-representational task, HFI. Notably, more than 80% of the ALS cohort exhibited deficits in visual-representational abilities (HFI), even at the early stages of the disease. These findings suggest that standard screening tools primarily focusing on basic visuo-perceptual skills may underestimate the extent of visuospatial deficits in ALS. On these bases, the common practice of assessing visuo-perceptual skills in ALS exclusively through screening tests might overlook visuospatial deficits typically linked to posterior cortical atrophy. This form of atrophy is commonly associated with the risk of cognitive deterioration in ALS and other neurodegenerative disorders, such as Parkinson’s disease [30].

The observed impairments likely result from neurodegenerative processes affecting the posterior parietal and frontal cortices, disrupting both elementary visuo-perceptual functions and more complex spatial cognition. Impaired performance on the LLJ task reflects deficits in spatial processing and attention, while difficulties on the LOJ task, which requires more intricate spatial orientation and mental visualization, are linked to dysfunction within the dorsal visual stream. Furthermore, HFI, which engages abstract reasoning and mental manipulation, underscores impairments in executive functions and working memory, consistent with the ALS-FTD continuum.

In contrast, no significant differences between patients with ALS and HCs were found in tasks such as AWJ and PPI, suggesting relative sparing of basic visuospatial abilities in ALS, particularly in tasks that impose minimal demands on motor or executive function. Likewise, higher-order visuospatial tasks like MR, CFI, and MC - which rely on the integrity of posterior brain regions - did not reveal significant impairments, supporting the notion that visuospatial deficits in ALS may be task-specific. These deficits appear to be more pronounced in domains that involve executive function, verbal fluency, and motor planning.

Building upon this, visuo-spatial impairments have been reported in several neurological disorders. In dementias, such as dementia with Lewy bodies, vascular dementia, and Alzheimer’s disease (AD), visuospatial deficits have been widely reported, although often neglected [31]. In fMRI studies on AD patients compared to HC, hypoactivation in visual task-related regions, such as the V5 area, the superior parietal lobe, the parieto-occipital cortex, and the premotor cortices have been observed in association with some compensatory hyperactivation in the inferior parietal lobule; these abnormalities were interpreted as the pathophysiological basis for visuospatial disorientation in patients with AD [32, 33]. In Parkinson’s disease, visual perception deficits are frequent and likely related to the potential pathophysiological role of basal ganglia and limbic structures in visuospatial functions [34].

In ALS, cognitive deficits are often reported in verbal fluency, language, social cognition, executive functions, and verbal memory, while visuospatial abilities appear to be less impaired [4]. Nonetheless, Boeve & Graff-Radford [35] found different degrees of impairment of cognitive abilities, including the visuospatial ones, in patients with *C9orf72* repeat expansions showing ALS and/or the frontotemporal dementia phenotype (c9FTD/ALS). In this subset of patients, in addition to bifrontal and cingulate cortex atrophy, structural MRI revealed parietal and occipital atrophy that could be part of the MRI signature pattern of c9FTD/ALS [36, 37]; this evidence might help explain the evidence of visuospatial dysfunction in this as well as in other phenotypes of ALS. Particularly, frontotemporal lobar degeneration with ubiquitin and TDP-43 positive neuronal inclusions may be associated with ALS, “progressive supranuclear palsy-like” syndrome, in which early behavioral disturbances, and marked visuospatial impairment is observed [38]. Nevertheless, Crockford and colleagues found no significant differences in visuospatial abilities across different disease stages, as assessed by ECAS [9]. However, lower cognitive abilities in ALS-specific functions and more behavioral alterations have been observed during the disease course. Probably, assessment tools more specific for detecting impairments in both components of visuospatial abilities, independent from motor disability, such as BVA, might reveal these cognitive dysfunctions in the early stages of the disease, suggesting the potential benefits of specific cognitive training protocols in ALS patients as well as in other neurological disorders [39, 40]. Indeed, from a clinical point of view, the integrity of visual and visuospatial abilities could play a pivotal role in using brain-computer interface (BCI) technology for improving communication abilities, assessing cognitive functions, and controlling external devices in patients with motor disabilities (for a review see [41]).

Although we obtained interesting insights into the visuospatial impairment in ALS, the generalization of the present findings is limited by the small sample size, the differences in cognitive assessments performed in the two studied groups, the lack of a validated composite disease severity index in ALS and the cross-sectional design of the study. Our small sample size allowed us to detect only large differences between the study groups and probably underestimated medium or small differences between the groups. Indeed, the small sample size may also partly explain the high variability of the confidence intervals. Future studies on larger samples are needed to better explore potential correlates of BVA with motor and non-motor factors and to consider the trends of correlations among Mental constructions / Hidden figure identification /Point Position Identification and UMN scores or ALSFRS-R. To note, we used the Bonferroni procedure, which may be too conservative given the small sample size. However, this procedure reduces the likelihood of false positives and supports the reliability of the present evidence. Moreover, an additional limit is the lack of inclusion of a positive-control group of subjects or a subset of patients carrying *C9orf72* repeat expansions. In this regard, neuroimaging studies on visuospatial impairments may provide valuable insights into their underlying mechanisms. For instance, Trojsi et al. [42] found that the reduction in neurovascular coupling within the default mode network (DMN) correlated with visuo-spatial ability, highlighting the link between neural activity and cognitive impairment in ALS, while Weil et al. [43] demonstrated that reduced functional connectivity in the DMN - specifically between the posterior cingulate cortex/precuneus and dorsomedial prefrontal cortex - predicts poor visuo-perceptual performance and cognitive decline in Parkinson’s disease. These findings highlight the potential of neuroimaging to elucidate the anatomical and functional correlates of visuospatial impairments in ALS as well as in other neurological conditions, underlining the need of further research to address this topic.

## Conclusions

The present study suggested an early impairment of visuospatial abilities in ALS, involving both perceptual and representational abilities, as assessed by BVA. In clinical practice, our findings provide new insight into multi-domain cognitive assessment in ALS to monitor disease progression effectively and organize care management properly. Further research on functional connectivity correlates of visuospatial functions might be important to better comprehend the impairment of extra-motor brain networks and address the dynamics of the spreading pathology in ALS.

## Electronic supplementary material

Below is the link to the electronic supplementary material.

Supplementary Material 1

## Data Availability

The datasets generated or analysed during the current study are available upon request to the corresponding author.

## Abbreviations

BVA: Battery for Visuospatial Abilities
ALSFRS-R: Amyotrophic Lateral Sclerosis Functional Rating Scale-Revised
ECAS: Edinburgh Cognitive and Behavioural ALS Screen
LLJ: Line Length Judgment
LOJ: Line Orientation Judgment
AWJ: Angle Width Judgment
PPI: Pointing Position Identification
MR: Mental Rotation
CFI: Complex Figure Identification
HFI: Hidden Figure Identification
MC: Mental Construction
