# Supplementary material for: Screening of visuospatial abilities in amyotrophic lateral sclerosis (ALS): a pilot study using the battery for visuospatial abilities (BVA)
Source: Orphanet J Rare Dis. 2025 Mar 8;20:110. doi: 10.1186/s13023-025-03645-z (PMC11890512; doi:10.1186/s13023-025-03645-z)
Supplement: Supplementary file 1 — Supplementary Material 1 [file 13023_2025_3645_MOESM1_ESM.docx]

**Supplementary Material 1.** Comparison between the subgroup of ALS free from impairments in executive functions and/or language disturbances (ALS) and healthy controls (HC) in age- and education-adjusted perceptive and representational tasks of BVA.

|  | ALS (*n*= 19) | HC (*n*= 23) | Mann-Whitney | *p*-value | Adj *p* |
| --- | --- | --- | --- | --- | --- |
| *BVA-perceptual tasks:* |  |  |  |  |  |
| Line length judgment (LLJ) | 15.86 [15.01, 17.03] | 18.66 [16.77, 19.06] | 74.50 | <0.001 | **<0.001** |
| Line orientation judgment (LOJ) | 6.22 [4.77, 8.12] | 8.55 [6.96, 9.27] | 121.00 | 0.014 | 0.109 |
| Angle width judgment (AWJ) | 2.04 [0.81, 4.33] | 4.90 [1.06, 5.46] | 128.00 | 0.022 | 0.176 |
| Point position identification (PPI) | 8.22 [7.69, 8.89] | 8.68 [7.02, 9.70] | 212.00 | 0.870 | 1.000 |
|  |  |  |  |  |  |
| *BVA-representational tasks:* |  |  |  |  |  |
| Mental rotation (MR) | 8.44 [7.63, 8.78] | 6.78 [3.30, 9.28] | 153.00 | 0.098 | 0.784 |
| Complex figure identification (CFI) | 8.19 [7.85, 8.43] | 7.76 [6.85, 8.22] | 152.50 | 0.095 | 0.760 |
| Hidden figure identification (HFI) | 0.00 [0.00, 1.61] | 3.52 [0.30, 5.22] | 90.00 | <0.001 | **<0.001** |
| Mental construction (MC) | 9.67 [4.24, 11.45] | 9.11 [5.18, 9.96] | 183.00 | 0.370 | 1.000 |

**Note.** Data are reported as median [25^th^ percentile, 75^th^ percentile]; Adj-*p* represents the *p*-value corrected for multiple comparisons using the Bonferroni procedure, and statistically significant differences are shown in **bold.**
